# Systemic effects of sunlight: 10‐year review of cardiovascular, infection and cancer outcomes

**DOI:** 10.1111/jdv.70461

**Published:** 2026-04-20

**Authors:** Jasira A. Ziglar, Rebecca L. Quiñonez, Lydiah Fridah M. Mpyisi, Indermeet Kohli, Yolanda Gilaberte, Henry W. Lim

**Affiliations:** ^1^ The Henry W. Lim Division of Photobiology and Photomedicine, Department of Dermatology Henry Ford Health Detroit Michigan USA; ^2^ University of Missouri School of Medicine Columbia Missouri USA; ^3^ Department of Medicine, College of Human Medicine Michigan State University East Lansing Michigan USA; ^4^ Department of Physics and Astronomy Wayne State University Detroit Michigan USA; ^5^ Department of Dermatology Miguel Servet University Hospital IIS Aragon Zaragoza Spain

**Keywords:** cardiovascular disease, mortality, sunlight, ultraviolet radiation

## Abstract

Sunlight is an important environmental determinant of human physiology, though its effects on health have been studied predominantly in the context of skin disease. While ultraviolet (UV) radiation is a well‐established risk factor for skin cancer, evidence suggests that it may also have beneficial systemic effects. This narrative review synthesizes evidence from the past decade on the associations between sunlight and UV radiation exposure and mortality‐related outcomes, with a focus on internal malignancies, cardiovascular disease and infectious diseases. A comprehensive search of PubMed and Embase was conducted for English‐language articles published between January 2015 and June 2025. Of 148 articles identified, two duplicates were removed. After title and abstract screening, 64 full‐text articles were assessed for eligibility; a total of 28 studies met the inclusion criteria and were included in the final review. Among the included studies, four examined cardiovascular outcomes, 12 evaluated infectious diseases, six focused on malignancies and six assessed all‐cause mortality. Overall, most studies reported inverse associations between UV exposure and adverse health outcomes, suggesting potential protective effects. For example, a large cohort study found that individuals with 2000 kJ/m^2^ higher annual solar radiation exposure had a 12% lower risk of all‐cause mortality. The comparator group had an average of 26 days of life lost over the 15.7‐year follow‐up period. This review identifies a pattern of potential beneficial associations between UV radiation exposure and systemic health outcomes, although confounding by various parameters including the healthy user effect and socioeconomic differences. When the benefits are considered alongside the well‐established deleterious cutaneous effects of UV exposure, these findings underscore the need for balanced public health messaging that acknowledges the risks and potential benefits of sunlight exposure.

## Graphical abstract

This review highlights the systemic effects of sunlight and ultraviolet radiation. While UV radiation exposure has known cutaneous risks including photoaging and skin cancer, evidence suggests potential associations with reduced all‐cause mortality, improved infectious disease outcomes and reduced blood pressure. These findings support balanced public health messaging.

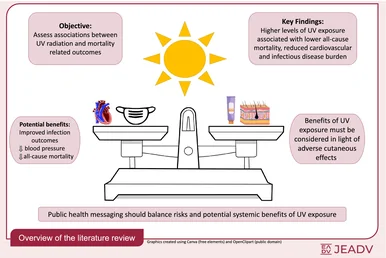

**Why was the study undertaken?**

- To explore the association between UV exposure and all‐cause mortality, with a focus on cardiovascular disease, infectious diseases and internal malignancies.

**What does this study add?**

- This review incorporates epidemiologic and clinical evidence examining the beneficial health outcomes associated with UV exposure, including a reduced risk of Hodgkin's lymphoma (OR 0.77, 95% CI 0.68–0.87) and non‐Hodgkin's lymphoma (OR 0.88, 95% CI 0.81–0.95), while acknowledging the well‐established adverse cutaneous effects.

**What are the implications of this study for disease understanding and/or clinical care?**

- The findings support the need for nuanced counselling that both acknowledges the cutaneous risks and possible systemic benefits of UV exposure.

## INTRODUCTION

Beyond its widely recognized biologic effects on the skin, sunlight is a key environmental determinant of human physiology. Although ultraviolet (UV) radiation is a well‐established risk factor for skin cancer, evidence suggests that sunlight and UV exposure have systemic health benefits.^1^

In addition to inducing vitamin D synthesis, UV radiation may improve health outcomes through other pathways. Some evidence shows that UVA exposure mobilizes nitric oxide stores in the skin, promoting vasodilation and lowering blood pressure, with implications for reduced cardiovascular risk.^2^, ^3^ Other proposed mechanisms include UV‐mediated immune modulation. UV‐induced protein fragments, known as neoantigens, may strengthen adaptive immunity by promoting B and T‐cell responses and increasing immune diversity, potentially improving resistance to infections and cancer development.^4^

Considering the growing interest in the systemic effects of UV exposure, this review synthesizes evidence from the past decade on the relationship between sunlight, UV radiation and mortality, with a particular focus on internal malignancies, cardiovascular outcomes and infectious diseases.

## MATERIALS AND METHODS

A comprehensive search of PubMed and Embase was conducted for English‐language articles published from January 2015 through June 2025, capturing 10.5 years of published research studies. Search terms included combinations of ultraviolet radiation, UV exposure, sunlight, solar radiation, mortality, internal cancer, cardiovascular disease and infection. The initial librarian‐assisted search resulted in 145 articles, with three additional studies added manually.

Of the 148 articles gathered, 2 duplicate records were removed. Two independent reviewers screened 146 titles and abstracts and 82 studies were excluded for lack of relevance, which included absence of UV, sunlight or mortality‐related outcomes. Sixty‐four full‐text articles were assessed for eligibility. Thirty‐six studies were excluded for the following reasons: 28 were review articles, 5 focused solely on skin cancer outcomes and 3 had insufficient assessment of sunlight or UV exposure. A total of 28 studies met the inclusion criteria and were incorporated into the final review.

## RESULTS

A total of 28 studies met the inclusion criteria. Study designs included ecological (16/28), cohort (8/28), cross‐sectional (1/28), case–control (1/28), case‐crossover (1/28) and meta‐analysis (1/28) (Tables 1, 2, 3, 4). Six studies evaluated the association between sunlight or UV radiation and all‐cause mortality, with most reporting inverse associations. Four studies investigated the cardiovascular effects of sunlight or UV radiation, overall supporting a potential protective association. Six studies evaluated internal malignancies in relation to UV radiation and sunlight exposure, with most studies pointing toward an inverse association, although the effect varied by organ system. Ten studies focused on COVID‐19 outcomes and generally showed reduced incidence and mortality with increased UV exposure. Exposure assessment varied substantially, with some studies using the UV Index, others using satellite‐derived data or self‐reported sun exposure.

**TABLE 1:** Effects of ultraviolet radiation on all‐cause mortality.

Author (year)	Country	Study population	Study design	Outcome measure	Key findings
Fu & Wang (2023)^5^	China	27 provinces	Ecological	Daily sunshine hours and crude mortality rate	Average daily sunshine duration ratio positively associated with higher provincial mortality
Nazeeh et al. (2025)^6^	The United States and Canada	83,205 adults	Cohort	Daylight exposure and mortality	Higher daylight exposure associated with lower all‐cause and noncancer non‐CVD mortality but no association with cancer mortality risk
Lindqvist et al. (2016)^7^	Sweden	29,518 women	Cohort	Sun exposure and all‐cause mortality	Low sun exposure associated with shorter life expectancy, dose‐dependent increased risk of CVD and noncancer non‐CVD deaths
Stevenson et al. (2024)^9^	The United Kingdom	>500,000 adults	Cohort	UV exposure and all‐cause and cause‐specific (CVD, cancer, non‐CVD/noncancer) mortality	Increased UV exposure associated with lower risk of all‐cause, CVD and cancer mortality
Salas et al. (2015)^10^	The United States	2319 infants	Cohort	UVB doses during fetal development and neonatal mortality rates	High‐intensity solar UVB exposure associated with lower mortality
Yoon et al. (2019)^8^	Korea	134,472 dialysis patients	Case‐crossover	Sunlight exposure and mortality of ESRD patient	Negative association between daily sunlight hours and all‐cause mortality in ESRD

Abbreviations: CVD, cardiovascular disease; ESRD, End‐stage renal disease.

### Overall mortality

Fu and Wang (2023) reported that a nonlinear transformation of average daily sunshine duration (modelled as the cubed term) was positively associated with provincial crude mortality rates in China; however, this finding was based on ecological data and did not specifically evaluate cause‐specific outcomes such as maternal mortality.^5^ Alternatively, data from the Adventist Health‐2 cohort study in Canada and the United States showed that more time spend outdoors during warmer months was associated with lower all‐cause mortality, cardiovascular disease (CVD) mortality and noncancer non‐CVD mortality.^6^ Notably, there was no significant association found between time spent outdoors and cancer mortality after adjusting for physical activity and other confounders.^6^ In a study by Lindqvist et al., women who avoided sun exposure had a lower life expectancy, largely due to increased risk of CVD and noncancer, non‐CVD deaths.^7^ Increased sun exposure was associated with reduced risk in these mortality categories.^7^ (Table 1).

Yoon et al. reported that end‐stage renal disease patients on dialysis with low sunlight exposure had higher all‐cause mortality, especially among older adults (>75 years) and those with diabetes.^8^ Stevenson et al. found that UV exposure in older adults in the United Kingdom was inversely associated with all‐cause, CVD and cancer mortality.^9^ Specifically, an increase in average annual shortwave radiation by 2000 kJ/m^2^ corresponded to a 12% lower risk of all‐cause mortality.^9^ (Table 1).

During fetal development, seasonal variations in solar UVB exposure were also associated with neonatal outcomes.^10^ Salas et al. found that higher UVB doses during weeks 17–22 of gestation were linked to lower neonatal mortality, whereas low UVB doses were not associated with increased mortality when compared with normal^10^ (Table 1).

### Cardiovascular effects

Sunlight and UV exposure appear to influence cardiovascular health and events (Table 2). Cannistraci et al. examined seasonal fluctuations in ST‐elevation myocardial infarction (STEMI) incidence and found that the typical predominance of daytime STEMI decreased during summer months, with some cohorts showing increased nocturnal STEMI.^11^ Similarly, Onozuka and Hagihara reported that 20% of the out‐of‐hospital cardiac arrests in Japan were attributable to daily solar radiation, primarily linked to low sunlight exposure.^12^

**TABLE 2:** Effects of ultraviolet radiation on cardiovascular outcomes.

Author (year)	Country	Study population	Study design	Outcome measure	Key findings
Cannistraci et al. (2018)^11^	China, Finland, Italy, Scotland, Japan, Australia, Singapore	2270 patients	Ecological	Seasonal variation in the circadian rhythm of STEMI onset	Difference between diurnal and nocturnal STEMI markedly decreased in the summer
Onozuka & Hagihara (2017)^12^	Japan	49,892 cases of OCHA	Ecological	Daily solar radiation and OCHA	Low solar radiation associated with a substantial attributable risk for OCHA
Lindqvist et al.(2021)^13^	Sweden	23,593 women	Cohort	Hypertension and sun exposure habits	Lower sun exposure associated with higher odds of hypertension
Weller et al. (2020)^14^	The United States	342,457 patients	Cohort	Incident solar UV radiation and blood pressure in haemodialysis patients	Negative linear association with pre‐dialysis systolic blood pressure

Abbreviations: OCHA, out of hospital cardiac arrest; STEMI, ST‐elevation myocardial infarction.

Levels of sun exposure may influence blood pressure. Lindqvist et al. observed that women with low and moderate sun exposure had 41% and 15% higher odds of hypertension, respectively, in comparison to those with high exposure.^13^ Weller et al. found that UV radiation exposure was associated with decreases in systolic blood pressure in haemodialysis patients, with stronger effects for UVB than UVA.^14^ This relationship persisted after adjusting for temperature.^14^

Though UV exposure may have positive effects on cardiovascular outcomes and all‐cause mortality, important confounding factors must be considered. While studies employed robust methodological approaches, including the use of independent and operationally distinct measures of sunlight exposure and adjustments for behavioural and socioeconomic factors, which together strengthen causal inference through triangulation, residual confounding cannot be fully excluded.^9^, ^13^ In particular, factors such as the timing and pattern of UV exposure, as well as the potential influence of additional lifestyle or environmental variables, may not have been fully captured and could still contribute to the observed associations.

### Cancer

The relationship between UV and sunlight exposure and cancer risk is complex and varies by cancer type (Table 3). Fleischer and Fleischer observed inverse associations between solar energy and incidence of invasive cancers, including brain, breast, colorectal, leukaemia, non‐Hodgkin's lymphoma, ovarian, prostate and urinary bladder cancers.^15^ In this analysis, keratinocyte carcinomas and melanomas were excluded. Conversely, solar energy was positively associated with cervix uteri and liver cancer incidence.^15^ Overall, solar energy appeared more strongly related to cancer incidence than mortality.^15^

**TABLE 3:** Effects of ultraviolet radiation on cancer risk.

Author (year)	Country	Study population	Study design	Outcome measure	Key findings
Fleischer & Fleischer (2016)^15^	The United States	48 states	Ecological	Average daily sunlight exposure and invasive cancer incidence	Higher solar energy inversely associated with GI, reproductive and haematologic cancers; positively with cervical and liver cancer
Zamoiski et al. (2016)^16^	The United States	36,725 participants	Ecological	Average lifetime UV exposure and breast cancer risk	No association between UV exposure and breast cancer risk.
Grant (2024)^17^	The United States	47 states	Ecological	UVB exposure and incident cancer rates	Solar UVB exposure associated with increased incidence of CNS, GI, renal/GU and haematological cancers
Rubenstein et al. (2017)^20^	The United States	166,795 oesophageal cancer deaths among non‐Hispanic white men in the United States	Ecological	Risk factors for geographical clustering of oesophageal cancer	Weak inverse association between UVR and oesophageal cancer mortality. Clustering patterns not explained by UV exposure.
Kim, Kim (2021)^18^	Multinational	NHL:216,285 HL:23,017 Data from 155 countries	Meta‐analysis (26 studies)	Sunlight exposure and lymphoid malignancy risk	Association between sunlight exposure and decreased risk of HL and NHL (except T‐cell)
Yu et al. (2025)^19^	China	2159 women who died from ovarian cancer and 2159 controls	Case–control	Outdoor ALAN and UVB radiation and ovarian cancer mortality	Dose–response relationship between ALAN exposure and ovarian cancer mortality risk

Abbreviations: ALAN, artificial light at night; CNS, central nervous systems; GI, gastrointestinal; GU, genitourinary; HL, Hodgkin's lymphoma; NHL, Non‐Hodgkin's lymphoma; UV, ultraviolet.

Studies of breast cancer specifically have shown inconsistent results. Zamoiski et al. reported no association between UV exposure (measured via ambient UVR, combined UVR or time outdoors) and breast cancer risk, with limitations including a predominantly white cohort and self‐reported sun exposure.^16^ Grant found that UVB exposure was associated with decreased incidence of bladder, brain (males), breast, oesophageal, non‐Hodgkin's lymphoma, corpus uteri, gastric, renal and pancreatic cancers in non‐Hispanic white populations.^17^

Evidence from a meta‐analysis of sunlight exposure and lymphoid malignancy suggests that UV exposure may be associated with a decreased lymphoma risk. Kim and Kim reported decreased risk of Hodgkin's lymphoma (OR 0.77, 95% CI 0.68–0.87) and non‐Hodgkin's lymphoma (OR 0.88, 95% CI 0.81–0.95), though heterogeneity between studies was high.^18^ For ovarian cancer, Yu et al. found no association between UVB exposure and risk, but artificial light at night was positively associated with ovarian cancer mortality.^19^ A geographic analysis of oesophageal cancer in the United States suggested a weak inverse association between UV exposure and mortality, though clustering patterns of increased disease mortality were not fully explained by UV or traditional oesophageal cancer risk factors.^20^

### Infections

UV radiation and sunlight may modulate infection‐related outcomes. During the COVID‐19 pandemic, multiple studies demonstrated inverse associations between sunlight exposure and COVID‐19 morbidity and mortality (Table 4). Higher ambient UVA and UVB exposures were linked to reduced COVID‐19 incidence and mortality in various countries, including the United States, Italy, the UAE, France and Spain.^21^, ^22^, ^23^, ^24^, ^25^, ^26^, ^27^, ^28^ Tang et al. further demonstrated that the UV dose from sunlight was negatively correlated with the average monthly per cent positivity of four common human coronaviruses (CoVHKU1, CoVNL63, CoVOC43 and CoV229E) in the United States using erythemal, vitamin D‐producing and DNA‐damage UV metrics from TEMIS datasets.^29^ A global analysis supports these findings. In a retrospective comparison of tropical and non‐tropical countries, Muhammad et al. observed significantly lower COVID‐19 case counts and mortality in regions with higher sunlight exposure.^30^

**TABLE 4:** Effects of ultraviolet radiation on infectious disease outcomes.

Author (year)	Country	Study population	Study design	Outcome measure	Key findings
Balcells et al. (2017)^31^	Chile	15 administrative regions	Ecological	SR and TB incidence/mortality/ hospital admission rates	Significant inverse association between SR and TB incidence, admission and mortality
Cherrie et al. (2021)^21^	The United States, England, Italy	The United States: 2474 counties England: 6724 output areas Italy: 6775 municipalities	Ecological	Ambient UVA radiation and COVID‐19 deaths	Higher ambient UVA exposure is associated with lower COVID‐19 mortality
Govani et al. (2015)^32^	The United States	45 states	Ecological	Average annual UV exposure and mortality in patients with CDI	Inverse relationship between inpatient mortality and average yearly UV index exposure in patients with CDI
Guasp, Laredo, Urra (2020)^22^	Multinational	359 countries	Ecological	Climatological factors and COVID‐19 incidence	Association between solar irradiance and reduced COVID‐19 incidence
Isaia et al. (2021)^23^	Italy	20 Italian regions	Ecological	Regional spatial distribution of COVID‐19 incidence and mortality	Association between UV exposure and geographical distribution of COVID‐19
Hachim et al. (2021)^24^	UAE	434 COVID‐19 positive patients	Ecological	Climatological factors and COVID‐19 outcomes	Admissions on days with higher solar radiation associated with less severe COVID‐19 outcomes
Muhammad et al. (2024)^30^	Multinational	221 countries (Tropical: 29 Non‐tropical: 125)	Ecological	Relative difference in COVID‐19 morbidity and mortality in tropical and non‐tropical countries by sunlight exposure	Inverse association between sunlight and COVID‐19 cases and mortality; lower rates in tropics
Moozhipurath et al. (2020)^27^	Multinational	152 countries	Ecological	Effect of UVB (using UVI as a proxy) on COVID‐19 deaths	Negative association between UVI and cumulative COVID‐19 deaths
Tang et al. (2021)^29^	The United States	50 states	Ecological	Per cent positive of 5 coronaviruses and UV radiation dose from sunlight	UV dose negatively correlated with the average per cent positivity of four human coronaviruses
Moozhipurath & Kraft (2021)^25^	Multinational	29,327 COVID‐19 deaths (155 countries)	Cohort	Association between lockdown severity and a reduction in the protective role of UVB exposure in COVID‐19	Increased lockdown severity was associated with decreased protective role of high UVI on COVID‐19 deaths
Perez‐Gilaberte et al. (2023)^26^	Spain	5 provinces	Cohort	COVID‐19 incidence ICU admissions and deaths and UVI	Inverse correlation between UVI and numbers of daily incident COVID‐19 cases
Lansiaux, et al. (2020)^28^	France	64,553,275 people	Cross‐Sectional	Sunlight exposure and COVID‐19 mortality rate	Average annual sunlight hours are significantly associated with COVID‐19 mortality rate

Abbreviations: CDI, clostridium difficile infection; ICU, intensive care unit; SR, solar radiation; TB, tuberculosis; UVA, ultraviolet A; UVB, ultraviolet B; UVI, ultraviolet index.

Beyond COVID‐19, UV exposure has been associated with outcomes in other infections. In Chile, Balcells et al. reported that lower solar radiation was linked to higher tuberculosis incidence, hospitalizations and mortality after adjusting for migration, HIV and incarceration rates.^31^ Similarly, Govani found that a higher mean annual UV Index was protective against mortality in patients hospitalized with *Clostridium difficile* infection in the United States.^32^

Many of the studies summarized are ecological, limiting inference at the individual level. Differences in data models for UV measurement, including UV Index, satellite‐derived UV data, ambient sunlight and self‐reported sun exposure, also affect comparability across studies.

## CONCLUSIONS

Evidence from studies of different designs and populations suggests that ultraviolet radiation and sunlight exert important effects on human health. Higher levels of UV exposure have been associated with lower all‐cause mortality, reduced cardiovascular and infectious disease burden and decreased incidence and mortality for several internal malignancies. At the same time, UV effects are not uniform across all organ systems, as cervix uteri and liver cancer demonstrate positive associations with solar exposure. These findings highlight the complexity of sunlight's biological influence.

Interpretation of the findings is limited by the ecological and observational nature of the included studies and potential confounding by geographic and socioeconomic factors. An additional limitation is the characterization of UV exposure, as many studies relied on ambient or outdoor UV levels as proxies. This may not translate into meaningful exposure of each individual, particularly in the presence of photoprotective behaviours. Despite these limitations, the overall pattern of evidence indicates that there are potential beneficial effects of UV exposure.

The benefits of UV exposure highlighted in this review must be considered in the context of its well‐established adverse cutaneous effects. The association between UV radiation and skin cancer is well‐established, with UV exposure serving as a primary cause of non‐melanoma skin cancer.^33^, ^34^, ^35^ Photoprotective measures, including seeking shade, wearing protective clothing, hats and applying sunscreens are essential in mitigating cutaneous damage.^36^ The findings of this review underscore important public health considerations, particularly the need for public education strategies that acknowledge both the risks and potential benefits of UV exposure in support of holistic public health promotion.

## AUTHOR CONTRIBUTIONS

HWL was involved with manuscript conception, drafting and final approval of the version to be published. JAZ, RLQ and LFM were involved in data analysis, drafting and revising the article. IK was involved in manuscript conception and drafting. YG was involved in critically revising the article.

## FUNDING INFORMATION

The authors have nothing to report.

## CONFLICT OF INTEREST STATEMENT

HWL has served as an investigator for Incyte, La Roche‐Posay, Pfizer and PCORI; as a consultant for ISDIN, Beiersdorf, Ferndale, L'Oréal, Eli Lilly, Zerigo Health, Skinosive, Kenvue, Cantabria Labs, NAOS and Boehringer Ingelheim; and as a speaker on general educational sessions for La Roche‐Posay, Cantabria Labs, Pierre Fabre, NAOS, Uriage, Pfizer, ISDIN, Clinuvel. YG has served as consultant for Almirall, ISDIN, Leo and Pfizer. She has received research grants from Sanofi and Almirall. She has participated as a speaker for Eucerin, NAOS–Bioderma, LEO Pharma, AbbVie, Sanofi, Lilly, La Roche‐Posay, ISDIN, Pierre Fabre and Cantabria Labs. IK has served as an investigator for Ferndale, Estée Lauder, La Roche‐Posay Dermatologique, Unigen, Johnson and Johnson, Allergan, Pfizer and Bayer; has received support from the American Skin Association for a vitiligo project; has served as a consultant for Pfizer, Johnson and Johnson, Beiersdorf (previously known as Bayer), La Roche‐Posay Dermatologique, Kenvue and ISDIN; and has received salary support from the Dermatology Foundation through a research career development award. JAZ, RLQ, LFM have no disclosures to declare.

## ETHICAL APPROVAL

Not applicable.

## ETHICS STATEMENT

This article is a literature review and did not involve human participants, animal subjects or the collection of primary data.

## Data Availability

Data sharing not applicable to this article as no datasets were generated or analysed during the current study.
